# Matrix Isolation FTIR and Theoretical Study of Weakly Bound Complexes of Isocyanic Acid with Nitrogen

**DOI:** 10.3390/molecules27020495

**Published:** 2022-01-13

**Authors:** Justyna Krupa, Maria Wierzejewska, Jan Lundell

**Affiliations:** 1 Faculty of Chemistry, University of Wrocław, Joliot-Curie 14, 50-383 Wrocław, Poland; maria.wierzejewska@chem.uni.wroc.pl; 2 Department of Chemistry, University of Jyväskylä, P.O. Box 35, FI-40014 Jyväskylä, Finland

**Keywords:** hydrogen bond, van der Waals interaction, vibrational spectroscopy, computational chemistry, molecular complex, atmospheric chemistry, HNCO

## Abstract

Weak complexes of isocyanic acid (HNCO) with nitrogen were studied computationally employing MP2, B2PLYPD3 and B3LYPD3 methods and experimentally by FTIR matrix isolation technique. The results show that HNCO interacts specifically with N_2_. For the 1:1 stoichiometry, three stable minima were located on the potential energy surface. The most stable of them involves a weak, almost linear hydrogen bond from the NH group of the acid molecule to nitrogen molecule lone pair. Two other structures are bound by van der Waals interactions of N⋯N and C⋯N types. The 1:2 and 2:1 HNCO complexes with nitrogen were computationally tracked as well. Similar types of interactions as in the 1:1 complexes were found in the case of the higher stoichiometry complexes. Analysis of the HNCO/N_2_/Ar spectra after deposition indicates that the 1:1 hydrogen-bonded complex is prevalent in argon matrices with a small amount of the van der Waals structures also present. Upon annealing, complexes of the 1:2 and 2:1 stoichiometry were detected as well.

## 1. Introduction

Non-covalent interactions are often involved in a variety of processes in biology and chemistry such as protein folding, DNA structure or molecular crystals formation. Weak molecular interactions have also an important contribution to chemical and physical processes taking place in the Earth’s atmosphere [1,2]. The vibrational properties of molecules taking part in such interactions appear to be very sensitive to the complexation. Therefore, infrared spectroscopy has been most often used to study various kinds of interactions, including van der Waals and hydrogen bonding. Among the most often studied weakly bound aggregates are those containing N_2_ molecules. Nitrogen, being the most abundant component of the Earth’s atmosphere is considered to be chemically inert. However, it has an electric quadrupole moment and was found to interact strongly with various molecules [3]. Such interaction leads to considerable changes in vibrational spectra of the complexed subunits, as demonstrated, for example, for nitrogen complexes isolated in low temperature matrices. For molecules with proton donor groups a weak traditional hydrogen bond of AH⋯N type (A=O, N, F, Cl, and others) is the most often encountered specific interaction [4,5,6,7,8,9,10,11,12,13,14,15,16,17,18,19,20,21,22]. Examples of the blue shifted hydrogen bonds can also be found in the literature, for instance, for the N_2_ complexes with N,N-dimethylformamide [23], chloroform [24] and difluoromethane [25]. Complexes of the noble gas hydrides with nitrogen have been shown to induce very large vibrational blue shifts for HXeCl⋯N_2_, HXeBr⋯N_2_ [26], HArF⋯N_2_, HKrF⋯N_2_, HKrCl⋯N_2_ [27] as well as for the (NgHNg)^+^ cations (Ng=Ar and Kr) [28]. Non-hydrogen bonded species formed between nitrogen and such molecules as dichlorosilylene SiCl_2_ [29], silylene SiH_2_ [30] or CO_2_ [31] have also demonstrated significant changes in the vibrational spectra of the complex subunits.

Isocyanic acid HNCO is an atmospheric pollutant that is emitted into the air from primary and secondary processes. The preliminary sources of this toxic compound are various combustion processes such as fossil fuel combustion and biomass burning [32]. In urban environments, HNCO can be found, for example, in tobacco smoke [33,34] and in vehicles’ exhaust [35,36,37]. The oxidation of atmospheric amines and amides are considered as secondary sources of HNCO as well [38,39]. Moreover, as a trace gas in ambient air, when absorbed while breathing, HNCO can participate in the protein carbamylation reactions, which are addressed in development of cardiovascular impairment, cataracts and rheumatoid arthritis [32]. In addition to research related to the chemistry of the atmosphere and in air, properties of HNCO and its isomers, as well as their photolysis channels, have been studied extensively both theoretically and experimentally [40,41,42,43,44,45,46,47,48,49,50,51,52,53,54,55,56,57,58]. However, there is much less data available on molecular complexes of HNCO [59,60,61,62,63], which would give insights on how the molecular properties and chemical reactivity of HNCO change upon intermolecular interactions.

In the present work, we report experimental results on the interaction of HNCO with nitrogen by using FTIR spectroscopy in an argon matrix. The experimental studies are supported by quantum chemical calculations at MP2, B2PLYPD3 and B3LYPD3 levels employing the 6-311++G(3df,3pd) and aug-cc-pVTZ basis sets. The properties and chemical reactivity of van der Waals and hydrogen bonded complexes of atmospheric constituents, especially those contributing to tropospheric and stratospheric chemistry, are worth studying. To our knowledge, complexes of isocyanic acid with nitrogen have not been the subject of either experimental or theoretical studies, and could be of interest for better understanding of interacting tropospheric gases.

## 2. Results and Discussion

### 2.1. Computational Results

Cartesian coordinates of all optimized species are provided in Table S1 in Supplementary Material. In general, the applied computational methods predicted similar geometries for the optimized complexes. The differences in the results for various computational approaches are commented upon below keeping the computational details to the minimum for the benefit of the experimental findings. In this paper, we present results of the MP2 [64,65,66,67], B2PLYPD3 [68,69,70] and B3LYPD3 [71,72,73,74,75] calculations using 6-311++G(3df,3pd) [76,77] basis set. Those obtained for aug-cc-pVTZ [78,79] basis set are available in Supplementary Material.

#### 2.1.1. Structure and Energetics of the 1:1 HNCO⋯N_2_ Complexes

**Figure 1:**
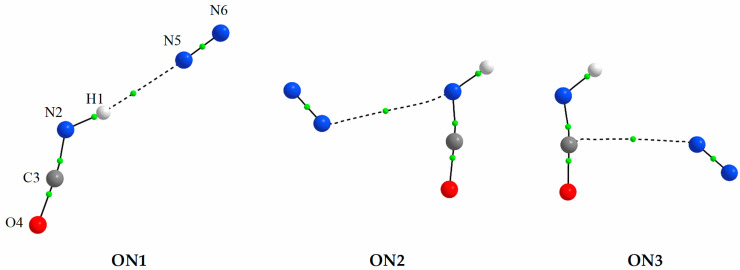
The MP2 optimized structures of the 1:1 HNCO⋯N_2_ complexes. The positions of the bond (3,−1) critical points derived from AIM calculations are shown by small green circles.

At the MP2 and B2PLYPD3 levels of theory three energy minima were found on the potential energy surface for the 1:1 HNCO complex with nitrogen. The structures related to these energy minima are shown in Figure 1 together with the adopted numbering. The first complex (ON1) is characterized by a weak, almost linear N-H⋯N hydrogen bond. Two other structures (ON2 and ON3) are bound through a weak van der Waals interaction. Interestingly, for a sulfur analogue of isocyanic acid, HNCS, two minima were located on the potential energy surface at MP2/6-311++G(2d,2p) level for interaction with nitrogen [17]. The first of them, which is the more stable of the two, strongly resembles that obtained for the HNCO⋯N_2_ complex, denoted ON1, whereas in the second one nitrogen molecule interacts with the sulfur atom of the HNCS moiety. There is no analogous structure on the potential energy surface for HNCO interaction with N_2_ to indicate a direct interaction with the oxygen atom. Instead, here the two other optimized structures (ON2 and ON3) depict the N-atom of the N_2_ molecule to interact with the N-atom or C-atom of the HNCO moiety. The most likely reason for this difference is a much higher computed Mulliken negative charge on the oxygen atom compared to that found on the sulfur atom in the HNCS molecule (−0.677 versus −0.081 at MP2/6-311++G(3df,3pd)) and double the positive charge on the carbon atom in HNCO compared to HNCS (1.460 versus 0.760).

AIM calculations were performed on the three HNCO⋯N_2_ complexes to scrutinize the type of interaction involved. Table 1 shows the MP2 calculated values of intermolecular distances and angles as well as values of two important topological AIM parameters: the electron density ρ(r) and its Laplacian ∇^2^ρ(r) at the bond critical points. These parameters are useful to determine the type of interaction in molecular systems. For the ON1 complex the values of both the electron density ρ(r) and its Laplacian ∇^2^ρ(r) at the BCP are in the proper ranges for hydrogen bonding of 0.0002–0.034 au and 0.024–0.139 au, respectively [80,81]. For the two other complexes, the ∇^2^ρ(r) values are smaller being outside the abovementioned interval, and thus indicating van der Waals interactions for ON2 and ON3.

Interestingly, using the B3LYPD3 methods combined with either applied basis sets led to the two stable minima ON1 and ON2, whereas the ON3 structure was not found to exist. Based on the structures shown in Figure 1, the ON3 structure indicates a C⋯N interaction, whereas the other structures indicate the N_2_ molecule interacts more directly to the nitrogen-containing end of HNCO. Table 2 and Table S2 (Supplementary Material) present the computed interaction energies for all 1:1 HNCO⋯N_2_ complexes, and based on the results acquired for the other computational levels, one could address this to a flat potential energy surface and negligible interaction energy well for B3LYPD3 calculations. A more rigorous study of the origin of this discrepancy between computational levels is outside the scope of this study. Alas, the discussion on energetics of the complexes is based primarily on the MP2 and B2PLYPD3 results.

All three ON1, ON2 and ON3 complexes are characterized by low interaction energies E_int_ in the range of 3.10–6.78 kJ mol^−1^ (MP2) and 2.59–6.61 (B2PLYPD3) kJ mol^−1^ (see Table 2 and Table S2). Among the three structures, the hydrogen bonded complex ON1 is the most stable one with the highest (the most negative) interaction energy. Out of the two van der Waals structures (ON2 and ON3) the more stable, at both levels of theory, is the ON3 in which the N5 atom of the nitrogen molecule interacts with the C-atom of the HNCO moiety. The ON2 form, in which N_2_ interacts with the N-atom of the acid, is the less stable of the two van der Waals complexes. It is worth noting that the order of stability is identical and the values of interaction energy E_int_ and relative energy ΔE obtained using both methods are very similar. However, as can be seen from Table 2 and Table S2, the order of the relative Gibbs free energy values ΔG does not follow the order of the relative energies ΔE, and in consequence, the less stable ON2 complex has a higher predicted abundance than the two other complex species. This observation is qualitatively adequate being based on room temperature calculations, which reflect thermal energies large enough to compete with the intermolecular interactions found in the complexes studied here. In the context of atmospheric chemistry and matrix isolation experiments employed here, which are connected with much lower temperatures, the interaction between the molecular subunits strongly favor the strongest existing interaction, the hydrogen-bonded ON1 structure. On the other hand, even though such complexes are short-lived and of low probability compared to the molecular subunits in the gas phase, they can contribute to the light-matter interaction, and provide additional channels for energy intake. For low temperature solid state chemistry, like ices, the probability of formation of such complexes increases, thereby affecting their photochemical participation.

#### 2.1.2. Structure and Energetics of the 1:2 and 2:1 HNCO⋯N_2_ Complexes

**Figure 2:**
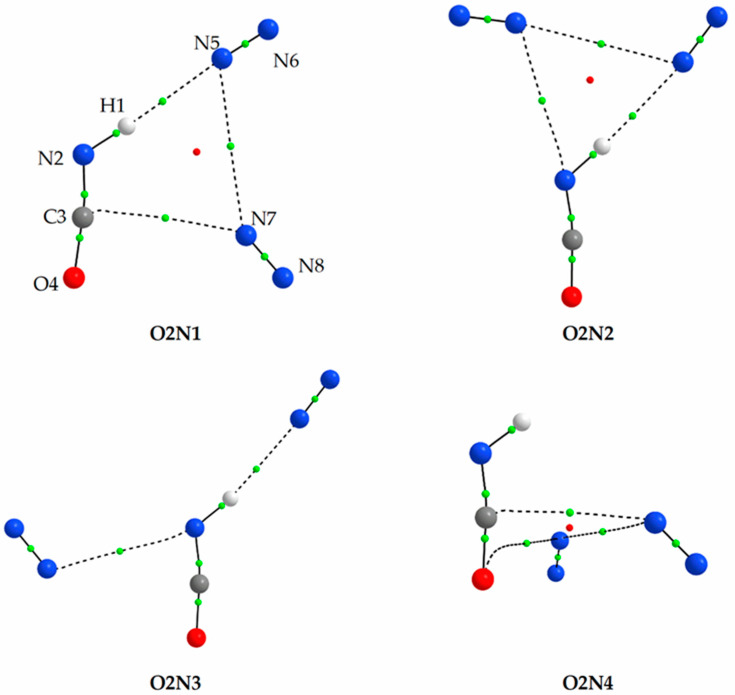
The MP2 optimized structures of the 1:2 HNCO complexes with N_2_. The positions of the bond (3,−1) and ring (3,+1) critical points derived from AIM calculations are shown by small green and red circles, respectively.

For HNCO interacting with two nitrogen molecules, four stable forms were located on the potential energy surface using the MP2 method. These structures are presented in Figure 2 and the MP2 calculated values of intermolecular distances and angles as well as values of two AIM parameters: the electron density ρ(r) and its Laplacian ∇^2^ρ(r) at the bond critical points are gathered in Table S3 in Supplementary Material. The analysis of the geometry of the 1:2 HNCO complexes with N_2_ shows, similarly to the 1:1 complexes, the presence of hydrogen and van der Waals intermolecular bonds. The calculated values of the AIM parameters suggest that these two types of the non-covalent interactions between the three moieties contribute to the stability of these ternary complexes. The presence of three bond critical points and their location reflect the appearance of the (3, +1) ring critical points due to the complex formation in O2N1, O2N2 and O2N4, indicating their consistent topology [80]. Similarly to the 1:1 complexes, not all MP2 optimized structures were found using DFT methods. Both B2PLYPD3 and B3LYPD3 failed to reproduce the O2N4 structure. According to the MP2 calculations, no hydrogen bond is present in the O2N2 structure, and the three components are bound only by intermolecular van der Waals interactions.

As shown in Table 3 and Table S4 in Supplementary Material, all optimized 1:2 complexes have similar interaction energies in the range of 9.00 to 12.43 kJ mol^−1^. The most stable structure for all employed computational levels is the O2N1 complex exhibiting a N-H⋯N hydrogen bond and a C⋯N van der Waals interaction. Points of attachment of N_2_ molecules in O2N1 are analogous to those in the 1:1 complexes ON1 and ON3.

**Figure 3:**
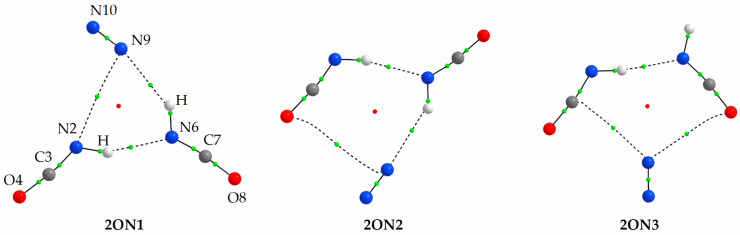
The MP2 optimized selected structures of the 2:1 HNCO complexes with N_2_. The positions of the bond (3,−1) and ring (3,+1) critical points derived from AIM calculations are shown by small green and red circles, respectively.

Ten structures were optimized for interaction of two HNCO molecules with N_2_ using MP2 method. Three of them, which are relevant to the experimental findings, are presented in Figure 3 and other structures are shown in Figure S1 in Supplementary Material. Data concerning energetics, geometry and AIM parameters of the 2:1 complexes are gathered in Table 4, Tables S5 and S6.

As it is seen in Figure 3 and Figure S1, interaction of two HNCO molecules with nitrogen leads to interesting structures. Most of them contain a HNCO dimer bound by N-H⋯N (2ON1, 2ON2 and 2ON3) or N-H⋯O (2ON5, 2ON6, 2ON7 and 2ON9) hydrogen bonds, and additional interaction of hydrogen bond or van der Waals type with the nitrogen molecule. Three other structures contain the HNCO dimer bound by C⋯O van der Waals interactions (2ON4, 2ON8 and 2ON10).

The 2:1 complexes are characterized by relatively similar interaction energy values between complex structures ranging from −26.02 to −16.48 kJ mol^−1^ (see Table 4 and Table S6). The three most stable structures, 2ON1, 2ON2 and 2ON3, presented in Figure 3, contain the N-H⋯N hydrogen bonded HNCO dimer interacting with the N_2_ molecule.

### 2.2. Matrix Isolation Infrared Spectra

A blank experiment was conducted for HNCO isolated in an argon matrix and the spectrum obtained agreed with those published in the literature [45,47]. It is interesting to note that the isocyanic acid monomer was found to rotate in an argon matrix [82]. In consequence, in the νNH stretching region of HNCO three bands due to the split rotational 0←1 transition are observed. Additionally, two components of the absorption due to the 0←0 transition originating from Fermi resonance are present. The unperturbed νNH fundamental in an argon matrix is at 3511.5 cm^−1^ [47]. The second intense band of monomeric HNCO, due to the asymmetric deformation mode ν_as_NCO, is located at 2259.0 cm^−1^. The other bands, arising from the δHNC and δNCO deformations, are characterized by relatively high intensity, and they should, in addition to the νNH and ν_as_NCO bands, serve as good markers providing information about the structure of the studied complexes [60]. Of the six infrared active modes of isocyanic acid, two vibrations, ν_s_NCO stretching and γNCO deformation, are expected to give rise to very weak bands [47].

The present analysis of the HNCO/N_2_/Ar spectra is based on changes observed in the νNH stretching region. Additionally, two other spectral regions are taken into account, namely asymmetric stretching ν_as_NCO and deformation δNCO. The remaining modes are either strongly coupled or give rise to very weak bands.

#### 2.2.1. HNCO⋯N_2_ Complexes of the 1:1 Stoichiometry

**Figure 4:**
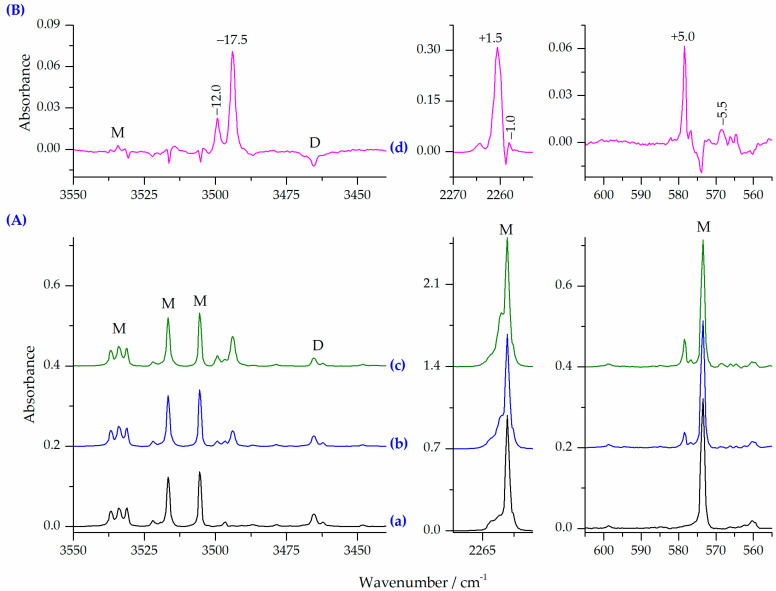
(**A**) The νNH, ν_as_NCO and δNCO regions in the spectra of matrices: HNCO/Ar = 1/6000 (a), HNCO/N_2_/Ar = 1/2/5600 (b), HNCO/N_2_/Ar = 1/4/5600 (c); (**B**) the difference spectrum (d) obtained by subtracting the spectrum (a) from the spectrum (c) (pink trace). Letters M and D denote the HNCO monomer and dimer bands, respectively.

When HNCO/Ar and N_2_/Ar mixtures were co-deposited at 15 K (10 K for measurements) several new bands were observed as compared with the HNCO/Ar spectrum. Figure 4 shows the νNH, ν_as_NCO and δNCO regions of the spectra of the HNCO/N_2_/Ar matrices obtained after deposition at two different HNCO/N_2_ ratios compared to the HNCO/Ar spectrum. The difference spectrum is presented in the upper part of the figure, as well showing new bands appearing upon complexation. In the spectra of the deposited HNCO/N_2_/Ar matrices (traces b and c in Figure 4), two new bands appeared in the νNH stretching region: the more intense of the two being at 3494.0 cm^−1^ and the second one at 3499.5 cm^−1^. The intensity ratio of these two bands is constant in the spectra independently on the concentration used. Thus, they are assigned to the 1:1 HNCO⋯N_2_ complexes. These bands are accompanied by new absorptions at 2260.5 and 578.5 cm^−1^ in the ν_as_NCO and δNCO regions, respectively. Based on the observed changes in the HNCO/N_2_/Ar spectrum, an attempt can be made to determine the structure of the 1:1 HNCO⋯N_2_ complexes formed in the matrices after deposition. Table 5 summarizes the selected wavenumber shifts calculated for the 1:1 complexes using the MP2, B2PLYPD3 and B3LYPD3 computational methods compared to the experimental results. In addition, theoretical infrared data and intensities obtained for the monomers and the 1:1 complexes are presented in Table S7 in Supplementary Material.

Comparison of the theoretical vibrational shifts with those found experimentally indicates that none of the applied methods reproduce the shifts observed in the HNCO/N_2_/Ar spectra. However, a set of bands with the shifts of −17.5, +1.5 and +5.0 cm^−1^ relatively well fits to the shifts predicted for the most stable structure ON1 (see Figure 1). Both νNH, ν_as_NCO are well reproduced whereas the shift of the δNCO mode is strongly overestimated. A possible explanation for this difference can be that the δNCO vibration is strongly coupled with the in-plane δHNC deformation mode. The second, weaker set of the observed bands with the shifts of −12.0, −1.0 and −5.5 cm^−1^ fits well to either of two less stable structures ON2 or ON3. Thus, in addition to the ON1 structure, one of the ON2 or ON3 structures is also present, in smaller amount, in the matrices obtained directly after deposition.

It is worth noting that an improvement of the agreement between the experimental and theoretical spectral results was obtained when the calculated shifts in the complexes were compared with those calculated for the corresponding HNCO complexes with an argon atom. These results are shown in Table S8 in Supplementary Material.

#### 2.2.2. HNCO⋯N_2_ Complexes of the 1:2 and 2:1 Stoichiometry

**Figure 5:**
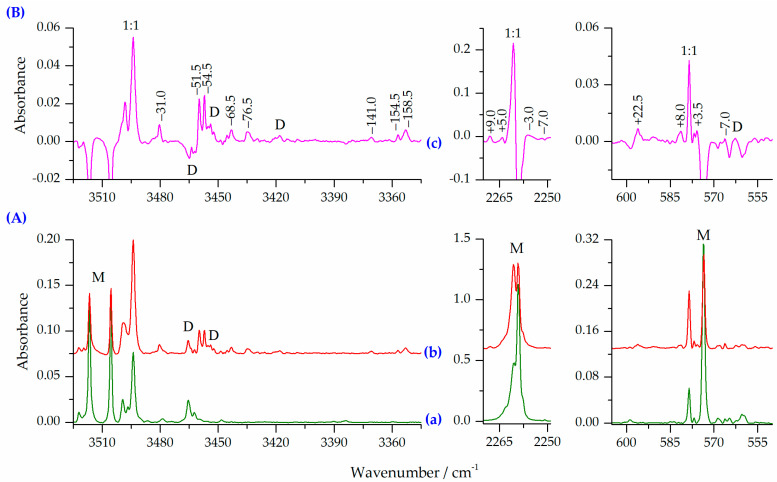
(**A**) The νNH, ν_as_NCO and δNCO regions in the spectra of matrices: HNCO/N_2_/Ar = 1/4/5600 (a), and matrix (a) after 10 min at 33 K/10 K (b); (**B**) the difference spectrum (c) obtained by subtracting the spectrum (a) from the spectrum (b) (pink trace). Letters M and D denote the HNCO monomer and dimer bands, respectively.

Figure 5 shows the νNH, ν_as_NCO and δNCO regions of the spectra of the HNCO/N_2_/Ar matrix obtained after deposition compared to the spectrum of the same matrix after annealing for 10 min at 33 K (10 K for measurement). The corresponding difference spectrum is also presented to show spectral changes upon annealing. Table 6 and Table 7 show the selected wavenumber shifts calculated for the 1:2 and 2:1 complexes using the MP2, B2PLYPD3 and B3LYPD3 methods compared to the experimental results. Additionally, the computed infrared wavenumbers, wavenumber shifts and intensities obtained for all the 1:2 and 2:1 complexes are presented in Tables S9 and S10 in Supplementary Material. The analysis of the HNCO/N_2_/Ar spectra obtained after annealing for 10 min at 33 K reveals that the amount of the 1:1 HNCO⋯N_2_ complex increases upon annealing. Simultaneously, many new bands appear, indicating that complexes with a higher stoichiometry are formed. These new bands are marked in the difference spectrum (Figure 5B) by showing the shifts compared to the corresponding HNCO monomer band positions.

Comparison of the experimental vibrational shifts with those predicted by computational methods allows us to assign these bands to both 1:2 and 2:1 complexes of HNCO with nitrogen. The position of two new bands appearing in the νNH and δNCO regions at 3480.5 and 596.0, with the shifts of −31.0 and +22.5 cm^−1^ fits very well to the shifts predicted for the three most stable HNCO complexes with N_2_ of the 1:2 stoichiometry, namely O2N1, O2N2 and O2N3. Since the calculated shifts of these modes are very similar for all these three structures, the obtained experimental spectra do not allow us to determine which of the 1:2 forms are present in the matrix after annealing. The fourth O2N4 complex is characterized by wavenumber shifts observed in the experimental spectra obtained upon deposition of the matrix (a set of bands with the −12.0, −1.0 and −5.5 cm^−1^ shifts) and assigned to one of the 1:1 species (ON2 or ON3) (see paragraph 2.2.1.). However, this set of bands does not increase their intensity on annealing, allowing us to exclude the O2N4 structure.

There are also bands in the spectra of the annealed HNCO/N_2_/Ar matrices which show larger shifts than those observed for the 1:2 complexes. The values of these shifts are shown in Table 7, and compared to the corresponding predicted values for the three selected 2:1 complexes. The corresponding shifts for other 2:1 geometries are summarized in Table S10 in Supplementary Material. The results collected in Table 7 show that annealing of the HNCO/N_2_/Ar matrices also leads to the formation of complexes with the 2:1 stoichiometry.

The analysis of the wavenumber shifts and intensities predicted for different 2:1 complexes allows us to indicate the three structures, which are most probably formed in the matrix upon annealing (see Figure 3). The presence of two acid molecules in such complexes determines that there are two vibrations of each type. The doublet band predicted for the νNH, ν_as_NCO and δNCO modes are characterized by distinctly different shifts. They are also expected to be of high intensity, with an exception of one of the δNCO modes. The bands identified in the experimental spectra and assigned to the 2:1 species are identified by the observed vibrational shifts very close to those predicted by computational methods. Similarly to the 1:2 stoichiometry structures, the vibrational shifts expected for the three considered structures are close to each other. However, since there are multiple bands observed in the spectra after annealing (see Figure 5), probably all three geometries of the 2:1 complexes are present in the matrices and it is not possible to unequivocally distinct them based on the existing data. Different structures are formed most likely in local relaxation processes, and the form of ternary complex is therefore deduced by the near vicinity distribution of complex subunits after deposition.

## 3. Experimental and Computational Details

### 3.1. Matrix Isolation FTIR Studies

Isocyanic acid, HNCO, was obtained by heating cyanuric acid powder (Acros Organics, 98%) at ca. T = 450 °C under vacuum in a quartz vessel. The HNCO vapor was condensed in a liquid-nitrogen trap, passed several times through P_2_O_5_ to remove traces of water and finally stored in a 250 mL glass bulb. The gaseous mixtures were prepared by mixing of HNCO and N_2_ (Messer, 6.0) with argon (Messer, 5.0) in two containers in a stainless steel vacuum system. Matrices were deposited through two jets onto a CsI window kept at 15 K. Pressure of the gas mixtures and the deposition rates were controlled by piezo transducers (model 902B, MKS Instruments, Uni-Export Instruments Polska, Warsaw, Poland) installed in both deposition lines. Low temperature was obtained using a closed cycle helium cryostat (APD-Cryogenics) and measured by a silicon diode sensor coupled with the digital controller (Scientific Instruments). Infrared spectra were taken at 10 K in a transmission mode with 0.5 cm^−1^ resolution by means of a Bruker IFS 66 Fourier Transform spectrometer (Bruker Polska Sp. z o.o., Poznań, Poland) equipped with a liquid cooled MCT detector.

### 3.2. Computational Methods

Computational studies for the 1:1, 1:2 and 2:1 complexes formed between HNCO and N_2_ were carried out using Gaussian16 program package [83]. Structures of monomers and complexes were optimized at the MP2 [64,65,66,67], B2PLYPD3 [68,69,70] and B3LYPD3 [71,72,73,74,75] levels of theory using the 6-311++G(3df,3pd) [76,77] and aug-cc-pVTZ [78,79] basis sets. The initial geometry of the 1:1 complexes was based on that found for HNCS⋯N_2_ [17]. The initial 1:2 and 2:1 structures were derived from the optimized 1:1 complexes and the structures of the HNCO dimer. Optimization of the complex structures was done with the Boys–Bernardi full counterpoise method by Dannenberg [84,85]. The interaction energies were estimated by subtracting the energies of the isolated monomers with the frozen geometry from the energy of the complexes. Vibrational wavenumbers and intensities were computed at the same levels using a harmonic approximation to confirm that the optimized structures correspond to the minima on the potential energy surfaces and to support the analysis of the experimental data. Spectral shifts upon complexation were obtained as the difference between the complex and monomer vibrational wavenumbers.

The topological analysis of the electron density (AIM) [86] was performed at the MP2/6-311++G(3df,3pd) level using AIM studio program (Version 19.10.12, Professional) [87], allowing us to characterize various types of interactions present in the aggregates.

## 4. Conclusions

For the first time, the results of theoretical and FTIR matrix isolation studies of weakly bound complexes formed between isocyanic acid and nitrogen are presented and discussed. The MP2 method revealed three, four and ten stable structures for HNCO complexes with N_2_ of the 1:1, 1:2 and 2:1 stoichiometry, respectively. Using the other two computational methods (B2PLYPD3 and B3PYPD3), a smaller number of the stable geometries was optimized for the 1:1 and 1:2 interactions (two and three, respectively). Based on the AIM results, it was shown that the interacting subunits are bonded either by the N-H⋯N hydrogen bond or by different van der Waals forces. Experimentally, HNCO complexes with N_2_ of three different stoichiometries were detected. Directly upon deposition of the matrix, the 1:1 species are present. Annealing at 33 K leads to the formation of higher aggregates HNCO with nitrogen of the 1:2 and 2:1 stoichiometry. Both experimental and computational studies indicate that HNCO and nitrogen molecules can engage into specific intermolecular interactions, leading to notable vibrational spectral changes. In the atmospheric and space chemistry context, such interactions could become important in low temperatures, and could induce additional energy intake channels in IR and UV/VIS photon energy regions in HNCO⋯N_2_ complexes and aggregates.

A number of nitrogen complexes with various proton donors has been previously studied in argon matrices. Table 8 shows the experimental relative shifts of the AH stretching mode in the AH⋯N_2_ hydrogen bonded complexes isolated in argon matrices. Comparison of the shifts indicates that isocyanic acid forms a complex with nitrogen of comparable strength to that of formic acid, but it is apparently weaker than the complexes of nitrogen with hydrogen fluoride and nitric acid. Additionally, this comparison indicates that HNCO⋯N_2_ is a plausible molecular complex in atmospheric contexts.

## Figures and Tables

**Table 1:** Interatomic distances (Å), angles (degree) and electron density parameters of the intermolecular bond critical points (au) of the HNCO complexes with N_2_ (1:1) computed at the MP2/6-311++G(3df,3pd) level.

Complex	Intermolecular Parameters ^1^			AIM Parameters		
	Interatomic Distances		Angle	BCP	ρ(r)	∇^2^ρ(r)
	H⋯Y	X⋯Y	X–H⋯Y			
ON1	4.372	6.258	170.7	H1⋯N5	0.0106	0.0419
ON2		6.187		N_2_⋯N5	0.0048	0.0197
ON3		5.992		C3⋯N5	0.0051	0.0215

^1^ X: N or C; Y: N.

**Table 2:** BSSE corrected interaction energies E_int_, relative energies ΔE, relative Gibbs free energies ΔG (kJ mol^−1^), abundance at 298 K A (%) and entropic contributions at 298 K TΔS (kJ mol^−1^) of the HNCO⋯N_2_ complexes of the 1:1 stoichiometry calculated at MP2, B2PLYPD3 and B3LYPD3 levels with the 6-311++G(3df,3pd).

Complex	MP2					B2PLYPD3					B3LYPD3				
	E_int_	ΔE	ΔG	A	TΔS ^1^	E_int_	ΔE	ΔG	A	TΔS	E_int_	ΔE	ΔG	A	TΔS
ON1	−6.53	0.00	4.72	9	−7.79	−6.49	0.00	5.40	7	−8.90	−6.40	0.00	4.51	14	−7.91
ON2	−3.10	3.40	0.00	61	0.00	−2.59	3.86	0.00	63	0.00	−2.68	3.72	0.00	86	0.00
ON3	−4.52	2.00	1.76	30	−3.11	−4.31	2.21	1.87	30	−3.44					

^1^ The calculated vibrational contribution to entropy is equal to (MP2) 86.58, 92.82, 90.32, (B2PLYPD3) 86.20, 93.32, 90.57 and (B3LYPD3) 86.87, 93.21 cal mol^−1^ K^−1^ for ON1, ON2 and/or ON3, respectively.

**Table 3:** BSSE corrected interaction energies E_int_ and relative energies ΔE (kJ mol^−1^) of the HNCO⋯N_2_ complexes of the 1:2 stoichiometry calculated at MP2, B2PLYPD3 and B3LYPD3 levels with the 6-311++G(3df,3pd) basis set.

Complex	MP2		B2PLYPD3		B3LYPD3	
	E_int_	ΔE	E_int_	ΔE	E_int_	ΔE
O2N1	−11.67	0.00	−11.13	0.00	−11.34	0.00
O2N2	−11.00	0.69	−10.17	0.98	−10.04	1.26
O2N3	−9.58	2.10	−9.04	2.08	−9.04	2.27
O2N4	−9.25	2.47				

**Table 4:** The BSSE corrected interaction energies E_int_ and relative energies ΔE (kJ mol^−1^) of the HNCO⋯N_2_ complexes of the 2:1 stoichiometry calculated at MP2, B2PLYPD3 and B3LYPD3 levels with the 6-311++G(3df,3pd) basis set.

Complex	MP2		B2PLYPD3		B3LYPD3	
	E_int_	ΔE	E_int_	ΔE	E_int_	ΔE
2ON1	−24.77	0.00	−24.60	0.00	−24.85	0.00
2ON2	−23.81	1.09	−23.97	0.71	−24.81	0.19
2ON3	−22.89	2.02	−22.64	2.07	−23.56	1.43
2ON4	−22.13	2.62	−22.72	1.86	−24.31	0.63
2ON5	−21.17	3.88	−22.13	2.70	−22.84	2.26
2ON6	−20.38	4.31	−20.67	3.83	−21.80	2.96
2ON7	−19.50	5.19	−19.66	4.84	−20.88	3.89
2ON8	−18.37	6.32	−18.70	5.79	−20.84	3.97
2ON9	−18.28	6.43	−18.62	5.90	−19.75	5.04
2ON10	−16.48	8.18	−17.03	7.44	−18.74	6.06

**Table 5:** Selected wavenumber shifts (cm^−1^) calculated for the 1:1 complexes using the MP2, B2PLYPD3 and B3LYPD3 methods with the 6-311++G(3df,3pd) basis set compared to the experimental results. The calculated intensities (km mol^−1^) of the bands are given in parentheses.

MP2			B2PLYPD3			B3LYPD3		Mode	Exp.^1^
ON1	ON2	ON3	ON1	ON2	ON3	ON1	ON2		
−25 (405)	−6 (169)	−9 (163)	−25 (401)	−5 (155)	−8 (149)	−23 (392)	−4 (155)	νNH	−17.5, −12.0
+2 (706)	−2 (631)	−2 (636)	+2 (732)	−1 (654)	−2 (654)	+2 (798)	0 (712)	ν_as_NCO	+1.5, −1.0
+21 (76)	−3 (83)	−6 (97)	+22 (63)	−3 (72)	−4 (81)	+20 (67)	−3 (75)	δNCO	+5.0, −5.5

^1^ The experimental shifts were calculated relative to the corresponding monomer band positions at 3511.5, 2259.0 and 573.5 cm^−1^, respectively.

**Table 6:** Selected wavenumber shifts (cm^−1^) calculated for the 1:2 complexes using the MP2, B2PLYPD3 and B3LYPD3 methods with the 6-311++G(3df,3pd) basis set compared to the experimental results. The calculated intensities (km mol^−1^) of the bands are given in parentheses.

MP2				B2PLYPD3			B3LYPD3			Mode	Exp.^1^
O2N1	O2N2	O2N3	O2N4	O2N1	O2N2	O2N3	O2N1	O2N2	O2N3		
−35 (380)	−33 (398)	−31 (397)	−11 (159)	−32 (376)	−31 (391)	−28 (394)	−29 (364)	−23 (345)	−26 (386)	νNH	−31.0
0 (662)	0 (740)	0 (661)	−1 (604)	0 (687)	0 (769)	0 (688)	0 (748)	0 (837)	+1 (751)	ν_as_NCO	
+16 (73)	+22 (81)	+19 (69)	−6 (96)	+17 (61)	+21 (68)	+19 (58)	+16 (66)	+16 (78)	+17 (62)	δNCO	+22.5

^1^ The experimental shifts were calculated relative to the corresponding monomer band positions at 3511.5, 2259.0 and 573.5 cm^−1^, respectively.

**Table 7:** Selected wavenumber shifts (cm^−1^) calculated for selected 2:1 complexes using the MP2, B2PLYPD3 and B3LYPD3 methods with the 6−311++G(3df,3pd) basis set compared to the experimental results. The calculated intensities (km mol^−1^) of the bands are given in parentheses.

MP2			B2PLYPD3			B3LYPD3			Mode	Exp.^1^
2ON1	2ON2	2ON3	2ON1	2ON2	2ON3	2ON1	2ON2	2ON3		
−64 (361)	−61 (340)	−39 (177)	−64 (367)	−60 (339)	−36 (163)	−60 (344)	−53 (315)	−34 (163)	νNH	−51.5, −54.5, −68.5, −76,5
−145 (769)	−150 (770)	−153 (784)	−154 (780)	−157 (762)	−158 (780)	−170 (830)	−174 (810)	−171 (808)		−141.0, −154.5, −158.5
+5 (486)	+5 (21)	+5 (697)	+7 (532)	+6 (32)	+7 (724)	+9 (635)	+8 (43)	+9 (799)	ν_as_NCO	+9.0, +5.0
−6 (1090)	−7 (1553)	−9 (650)	−7 (1103)	−8 (1599)	−10 (679)	−7 (1145)	−8 (1738)	−9 (734)		−3.0, −7.0
+66 (0)	+63 (37)	+61 (22)	+61 (1)	+62 (2)	+57 (2)	+64 (2)	+65 (3)	+61 (4)	δNCO	n.o.
+4 (187)	+11 (26)	−5 (72)	+9 (145)	+13 (20)	−3 (60)	+10 (137)	+14 (22)	−1 (60)		+8.0, +2.5, −7.0

^1^ The experimental shifts were calculated relative to the corresponding monomer band positions at 3511.5, 2259.0 and 573.5 cm^−1^, respectively.

**Table 8:** The relative shifts observed for hydrogen bonded complexes of HA with nitrogen isolated in argon matrices.

Proton Donor HA	Relative Shifts (%) ^1^	Reference	Proton Donor HA	Relative Shifts (%)	Reference
HF	0.99	[4]	HNCO	0.50	This work
HNO_3_	0.97	[14]	HNCS	0.46	[17]
CF_3_COOH	0.91	[15]	HONO*-trans*	0.34	[13]
H_2_SO_4_	0.56	[16]	HCl	0.27	[88]
HCOOH	0.53	[11]	CH_3_OH	0.19	[9]

^1^ The relative shifts were calculated as ΔνAH/νAH⋯N_2_.

## Data Availability

Data is contained within the article or the supplementary material. The data presented in this study are available.

## Supplementary Materials

The following supporting information can be downloaded online, Figure S1: The MP2 optimized selected structures of the 2:1 HNCO complexes with N_2_. The positions of the bond (3,−1) and ring (3,+1) critical points derived from AIM calculations are shown by small green and red circles, respectively; Table S1: The MP2 cartesian coordinates of all 1:1, 1:2 and 2:1 optimized species of HNCO with N_2_; Table S2: BSSE corrected interaction energies E_int_, relative energies ΔE, relative Gibbs free energies ΔG (kJ mol^−1^), abundance at 298 K A (%) and entropic contributions at 298 K TΔS (kJ mol^−1^) of the HNCO⋯N_2_ complexes of the 1:1 stoichiometry calculated at MP2, B2PLYPD3 and B3LYPD3 levels with the aug-cc-pVTZ basis set; Table S3: Interatomic distances (Å), angles (degree) and electron density parameters of the intermolecular bond critical points BCP (au) and ring critical points RCP(au) of the HNCO complexes with N_2_ (1:2) computed at the MP2/6-311++G(3df,3pd) level; Table S4: BSSE corrected interaction energies E_int_ and relative energies ΔE (kJ mol^−1^) of the HNCO⋯N_2_ complexes of the 1:2 stoichiometry calculated at MP2, B2PLYPD3 and B3LYPD3 levels with the aug-cc-pVTZ basis set; Table S5: Interatomic distances (Å), angles (degree) and electron density parameters of the intermolecular bond critical points BCP (au) and ring critical points RCP(au) of the HNCO complexes with N_2_ (2:1) computed at the MP2/6-311++G(3df,3pd) level; Table S6: BSSE corrected interaction energies E_int_ and relative energies ΔE (kJ mol^−1^) of the HNCO⋯N_2_ complexes of the 2:1 stoichiometry calculated at MP2, B2PLYPD3 and B3LYPD3 levels with the aug-cc-pVTZ basis set; Table S7: Theoretical infrared wavenumbers (ῡ, cm^−1^), wavenumber shifts (Δῡ, cm^−1^) and intensities (I, km mol−1) for monomers and 1:1 complexes using the MP2, B2PLYPD3 and B3LYPD3 methods with basis sets 6-311++G(3df,3pd) and aug-cc-pVTZ; Table S8: Selected wavenumber shifts (cm^−1^) calculated for the 1:1 complexes using the MP2, B2PLYPD3 and B3LYPD3 methods with the 6-311++G(3df,3pd) basis set. The shifts were calculated relative to the values obtained for the corresponding HNCO⋯Ar complexes. The calculated intensities (km mol−1) of the bands are given in parentheses; Table S9: Theoretical infrared wavenumbers (ῡ, cm^−1^), wavenumber shifts (Δῡ, cm^−1^) and intensities (I, km mol−1) for 1:2 complexes using the MP2, B2PLYPD3 and B3LYPD3 methods with basis sets 6-311++G(3df,3pd) and aug-cc-pVTZ; Table S10: Theoretical infrared wavenumbers (ῡ, cm^−1^), wavenumber shifts (Δῡ, cm^−1^) and intensities (I, km mol−1) for 2:1 complexes using the MP2, B2PLYPD3 and B3LYPD3 methods with basis sets 6-311++G(3df,3pd) and aug-cc-pVTZ.

## Sample Availability

Samples of the compounds are not available from the authors.
