# Supplementary material for: Matrix Isolation FTIR and Theoretical Study of Weakly Bound Complexes of Isocyanic Acid with Nitrogen
Source: Molecules. 2022 Jan 13;27(2):495. doi: 10.3390/molecules27020495 (PMC8777744; doi:10.3390/molecules27020495)
Supplement: Supplementary file 1 [file molecules-27-00495-s001.zip › molecules-1540796-supplementary.pdf]

Supplementary Materials

Matrix isolation FTIR and theoretical study of weakly bound complexes of isocyanic acid with nitrogen

Justyna Krupa, Maria Wierzejewska, Jan Lundell

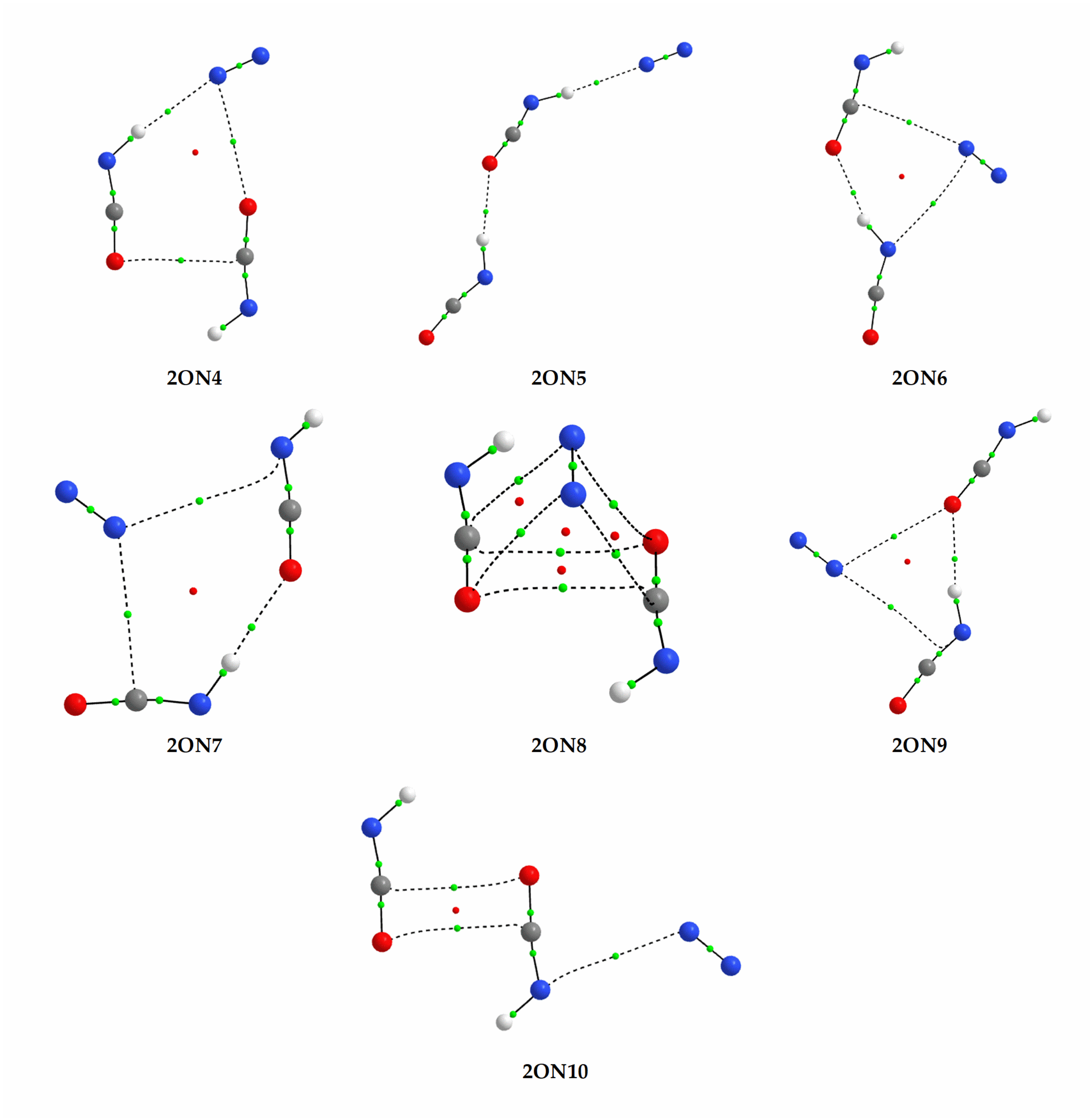

Figure S1. The MP2 optimized selected structures of the 2:1 HNCO complexes with N_2. The positions of the bond (3,-1) and ring (3,+1) critical points derived from AIM calculations are shown by small green and red circles, respectively.

Table S1. The MP2/6-311++G(3df,3pd) cartesian coordinates of all 1:1, 1:2 and 2:1 optimized species of HNCO with N_2.

1:1

ON1

0 1

N	-3.46376200	-0.21488900	-0.00000300
N	-2.36142600	-0.06317300	0.00000300
N	0.87662100	0.73274300	0.00000000
C	1.88047000	0.04407700	0.00000000
H	-0.06742700	0.38540200	0.00000500
O	2.92807200	-0.47907800	-0.00000100

ON2

0 1

N	-1.10273400	1.20037000	0.00000200
C	-1.46938800	0.03658700	0.00000100
H	-1.73182700	1.98419000	-0.00000100
O	-1.66295600	-1.11645700	0.00000000
N	1.78287000	-0.41431300	-0.00000500
N	2.72726500	0.17507700	0.00000300

ON3

0 1

H	-1.06175500	-1.82360900	0.00000100
N	-1.72549600	-1.06851000	-0.00000200
C	-1.39953700	0.10616200	-0.00000100
O	-1.24412400	1.26599400	0.00000100
N	2.72301500	0.18776300	-0.00000500
N	1.77561900	-0.39658400	0.00000700

1:2

O2N1

0 1

N	3.44171500	-0.42994800	-0.00006500
N	2.39083500	-0.79618900	0.00005100
N	-0.80588600	-1.75893300	0.00029700
C	-1.76950900	-1.01476900	0.00008000
H	0.15008700	-1.44322100	0.00026300
O	-2.78402200	-0.42930300	-0.00009600
N	-0.27769800	2.82289000	-0.00016400
N	-0.07194700	1.72878900	-0.00011600

O2N2

0 1

N	-2.38537800	-2.36996100	-0.00033100
N	-1.42126400	-1.81421900	-0.00022600
N	1.20517700	0.23678100	0.00015200
C	2.38613300	-0.05999200	0.00011300
H	0.44125700	-0.41856400	0.00001700
O	3.55032700	-0.18570400	0.00010600
N	-1.77936000	1.57884500	0.00000200
N	-1.78498500	2.69200400	0.00018300

O2N3

0 1

N	-4.41385700	-0.77365300	-0.00001400
N	-3.35458200	-0.43288400	-0.00000600
N	-0.08932700	0.24904700	0.00003200
C	0.51261400	1.30856100	0.00001800
H	-1.09159700	0.15838700	0.00001600
O	1.22407200	2.23774700	0.00001000
N	3.13477300	-1.92671400	0.00009000
N	3.04061300	-0.81747100	-0.00013000

O2N4

0 1

N	0.64282800	1.93913800	-0.31900700
N	1.48236400	2.37282600	0.26963900
N	-2.16569000	-0.16614800	-0.93790400
C	-1.67224000	-0.23644000	0.17416900
H	-1.70907400	0.28484500	-1.71202000
O	-1.33120500	-0.38379900	1.28425500
N	1.28426600	-1.42251100	-0.46608500
N	1.95511000	-2.12270600	0.08092400

2:1

2ON1

0 1

H	0.45405700	-0.57924400	-0.00001300
N	1.45216800	-0.41243800	0.00000600
C	2.32126600	-1.26386300	0.00004900
O	3.26061600	-1.96450600	0.00009100
H	-1.51994600	0.89923100	-0.00003400
N	-1.58180500	-0.10778200	-0.00003100
C	-2.64000800	-0.72028800	-0.00001200
O	-3.56477100	-1.43178600	0.00000400
N	0.89131600	3.36894100	-0.00005900
N	0.01140300	2.68774400	-0.00004900

2ON2

0 1

H	0.16298300	-1.37838600	0.00096200
N	-0.70280200	-1.90301800	0.00118600
C	-1.81858100	-1.42252600	-0.00000400
O	-2.94574100	-1.09526000	-0.00097500
H	1.10332400	0.99266000	0.00022000
N	1.61856000	0.12547300	0.00028900
C	2.84084300	0.07269600	-0.00016400
O	3.98961200	-0.12917000	-0.00054100
N	-1.06467300	1.99178600	0.00013300
N	-2.10120600	2.39720800	0.00009900

2ON3

0 1

H	0.42432600	-1.51419400	-0.00006700
N	1.42849400	-1.64368900	-0.00013800
C	2.24961600	-0.74714800	-0.00000100
O	3.14625000	0.00901800	0.00010300
H	-2.04355100	-2.49928600	0.00009700
N	-1.66478700	-1.56564400	0.00008100
C	-2.38339800	-0.57405300	0.00018800
O	-2.93830200	0.45130600	0.00027600
N	0.08273100	1.64511800	-0.00007200
N	0.26189400	2.74394200	-0.00046800

2ON4

0 1

H	-3.13553600	-1.17663100	0.00026200
N	-2.37057500	-1.83050500	0.00016700
C	-1.20345500	-1.49015200	0.00003500
O	-0.04382600	-1.31279700	-0.00010000
H	1.15213600	1.59352800	-0.00006700
N	0.32599300	2.17036400	0.00000500
C	-0.81223100	1.74861900	0.00008300
O	-1.95689100	1.48686500	0.00016400
N	2.83905700	0.03649000	-0.00016200
N	3.50313200	-0.85638200	-0.00021200

2ON5

0 1

H	-2.65779100	0.59592400	0.19669700
N	-1.86800500	1.19469300	0.36724400
C	-0.73356100	1.06889300	-0.03577200
O	0.39869800	1.08824800	-0.34861700
H	2.11864900	-0.03697200	-0.06576100
N	2.84827600	-0.71232600	0.10292100
C	4.04457700	-0.50727100	0.03160500
O	5.21558000	-0.46333700	0.00331900
N	-4.59818800	-0.60091100	-0.01001100
N	-5.55939300	-1.15688000	-0.08066100

2ON6

0 1

H	-0.81615800	-0.73221800	0.11447800
N	-1.58050900	-0.09043000	-0.03173500
C	-2.76122000	-0.38460800	-0.05266100
O	-3.92478300	-0.51186500	-0.09926700
H	3.69973600	0.01851500	-0.37831900
N	3.30075800	-0.89880700	-0.28652600
C	2.15607300	-1.14031300	0.03348300
O	1.09228400	-1.53815900	0.32935500
N	1.15263400	1.93182100	0.02118500
N	0.47101600	2.80933300	0.08824800

2ON7

0 1

H	-3.75512200	0.14619900	1.04123300
N	-3.05842100	0.18097000	0.31887400
C	-2.29017500	-0.70766100	0.01453900
O	-1.49239500	-1.46244700	-0.39761400
H	0.58687800	-1.64202100	-0.16965800
N	1.57836100	-1.66094800	0.01485100
C	2.29121300	-0.67911800	0.09467900
O	3.09524500	0.16793300	0.19969800
N	0.07639000	2.73654200	-0.18380500
N	0.02355800	1.62523800	-0.14185500

2ON8

0 1

H	2.08756000	-0.66164700	1.54058800
N	2.37080300	-0.69268200	0.57572100
C	1.59092800	-0.73604800	-0.35547600
O	0.96334100	-0.77288000	-1.34575000
H	-2.08798300	-0.66078600	-1.54057000
N	-2.37125700	-0.69161800	-0.57570000
C	-1.59139800	-0.73527100	0.35550100
O	-0.96385100	-0.77227900	1.34579300
N	0.30666200	2.30004200	0.46531200
N	-0.30516300	2.30020300	-0.46540500

2ON9

0 1

H	-4.56142600	-1.05834400	-0.27067400
N	-3.91438300	-0.46977300	0.22197500
C	-2.74516200	-0.29084900	-0.04438200
O	-1.61240900	-0.01060300	-0.17082600
H	0.24545400	-1.00265100	-0.07635700
N	1.04891100	-1.61217500	-0.05853100
C	2.20013400	-1.22308900	0.01196700
O	3.34895800	-1.00193800	0.07615000
N	0.88128400	2.96294900	0.00305300
N	1.08329500	1.86827700	0.01906500

2ON10

0 1

H	-2.80368400	-1.95745100	0.00001500
N	-3.36619200	-1.12337300	-0.00004400
C	-2.90131100	0.00002400	-0.00003000
O	-2.60055300	1.13357400	-0.00003100
H	0.24427400	2.16040200	0.00003800
N	0.81173500	1.32912600	0.00004300
C	0.34200000	0.20648500	0.00005600
O	0.03434600	-0.92447800	0.00006900
N	3.52237000	-0.56803400	0.00003600
N	4.52422000	-0.08268600	-0.00010700

Table S2. BSSE corrected interaction energies E_{int}, relative energies ΔE, relative Gibbs free energies ΔG (kJ mol^{-1}), abundance at 298 K A (%) and entropic contributions at 298 K $T\Delta S$ (kJ mol^{-1}) of the HNCO···N_2 complexes of the 1:1 stoichiometry calculated at MP2, B2PLYPD3 and B3LYPD3 levels with the aug-cc-pVTZ basis set.

Complex	MP2					B2PLYPD3					B3LYPD3				
	E_{int}	ΔE	ΔG	A	TΔS[1]	E_{int}	ΔE	ΔG	A	TΔS	E_{int}	ΔE	ΔG	A	TΔS
ON1	-6.78	0.0	4.64	9	-7.67	-6.61	0.00	5.15	8	-8.68	-6.44	0.00	4.37	15	-7.79
ON2	-3.39	3.4	0.00	61	0.00	-2.68	3.90	0.00	62	0.00	-2.68	3.75	0.00	85	0.00
ON3	-4.85	1.9	1.82	30	-3.23	-4.39	2.22	1.79	30	-3.40					

[1] The calculated vibrational contribution to entropy is equal to (MP2) 86.43, 92.57, 89.99, (B2PLYPD3) 86.15, 93.11, 90.39 and (B3LYPD3) 86.85, 93.10 cal mol^{-1} K^{-1} for ON1, ON2 and/or ON3, respectively

Table S3. Interatomic distances (Å), angles (degree) and electron density parameters of the intermolecular bond critical points BCP (au) and ring critical points RCP(au) of the HNCO complexes with N_2 (1:2) computed at the MP2/6-311++G(3df,3pd) level.

Complex	Intermolecular parameters[1]			AIM parameters		
	Interatomic distances		Angle	BCP	ϱ(r)	∇²ϱ(r)
	H···Y	X···Y	X–H···Y			
O2N1	4.407	6.309	177.8	H1···N5	0.0100	+0.0392
		6.097		C3···N7	0.0046	+0.0183
		6.665		N5···N7	0.0025	+0.0093
			RCP	(5 at.)	0.0019	+0.0082
O2N2	4.346	6.242	173.8	H1···N5	0.0109	+0.0429
		6.137		N2···N7	0.0043	+0.0185
		6.403		N5···N5	0.0032	+0.0129
			RCP	(4 at.)	0.0023	+0.0098
O2N3	4.420	6.304	170.5	H1···N5	0.0099	+0.0388
		6.249		N2···N7	0.0047	+0.0167
O2N4		5.998		C3···N5	0.0050	+0.0212
		6.200		O4···N7	0.0047	+0.0204
		6.435		N5···N7	0.0030	+0.0119
			RCP	(4 at.)	0.0020	+0.0086

[1] X: N,O or C
Y: N

Table S4. BSSE corrected interaction energies E_{int} and relative energies ΔE (kJ mol^{-1}) of the HNCO···N_2 complexes of the 1:2 stoichiometry calculated at MP2, B2PLYPD3 and B3LYPD3 levels with the aug-cc-pVTZ basis set.

Complex	MP2		B2PLYPD3		B3LYPD3	
	E_{int}	ΔE	E_{int}	ΔE	E_{int}	ΔE
O2N1	-12.43	0.0	-11.38	0.00	-11.34	0.00
O2N2	-11.59	0.8	-10.42	0.96	-10.13	1.20
O2N3	-10.13	2.2	-9.25	2.11	-9.08	2.25
O2N4	-10.04	2.4				

Table S5. Interatomic distances (Å), angles (degree) and electron density parameters of the intermolecular bond critical points BCP (au) and ring critical points RCP(au) of the HNCO complexes with N_2 (2:1) computed at the MP2/6-311++G(3df,3pd) level.

Complex	Intermolecular parameters[1]			AIM parameters		
	Interatomic distances		Angle	BCP	ϱ(r)	∇²ϱ(r)
	H···Y	X···Y	X–H···Y			
**2ON1**	3.949	5.762	157.5	H1···N6	0.0187	+0.0636
	4.449	6.080	142.9	H5···N9	0.0099	+0.0399
		6.460		N2···N9	0.0033	+0.0117
			**RCP**	(5 at.)	0.0024	+0.0113
**2ON2**	2.093	3.083	165.3	H1···N6	0.0190	+0.0621
	2.387	3.268	145.4	H5···N9	0.0092	+0.0368
		3.615		O4···N9	0.0028	+0.0108
			**RCP**	(7 at.)	0.0017	+0.0075
**2ON3**	3.949	5.847	171.2	H1···N6	0.0190	+0.0617
		6.100		C3···N9	0.0045	+0.0179
		6.138		O8···N9	0.0046	+0.0179
			**RCP**	(7 at.)	0.0022	+0.0092
**2ON4**	4.338	6.230	172.2	H1···N9	0.0110	+0.0424
		6.015		O8···N9	0.0042	+0.0174
		5.803		O4···C7	0.0066	+0.0258
			**RCP**	(7 at.)	0.0023	+0.0106
**2ON5**	3.921	5.808	170.3	H1···O8	0.0156	+0.0643
	4.326	6.216	172.9	H5···N9	0.0110	+0.0431
**2ON6**	3.936	5.784	162.8	H1···O8	0.0154	+0.0639
		6.426		N2···N9	0.0037	+0.0125
		6.107		C7···N9	0.0047	+0.0189
			**RCP**	(5 at.)	0.0019	+0.0089
**2ON7**	3.967	5.867	174.2	H1···O8	0.0150	+0.0602
		6.490		N6···N9	0.0041	+0.0158
		6.126		C3···N9	0.0043	+0.0180
			**RCP**	(7 at.)	0.0021	+0.0093
**2ON8**		5.801		C3···O8	0.0066	+0.0273
		5.801		O4···C7	0.0066	+0.0273
		6.420		C3···N9	0.0033	+0.0132
		6.499		O8···N9	0.0034	+0.0131
		6.499		O4···N10	0.0034	+0.0131
		6.420		C7···N10	0.0033	+0.0132
			**RCP** (OCOC)	(4 at.)	0.0066	+0.0277
			**RCP** (OCNN)	(4 at.)	0.0033	+0.0142
			**RCP** (CONN)	(4 at.)	0.0033	+0.0142
			**RCP**	(6 at.)	0.0020	+0.0096
**2ON9**	3.984	5.873	170.8	H1···O8	0.0144	+0.0596
		6.579		N2···N9	0.0047	+0.0172
		6.220		O8···N9	0.0034	+0.0139
			**RCP**	(4 at.)	0.0025	+0.0117
**2ON10**		5.816		C3···O8	0.0065	+0.0266
		5.830		O4···C7	0.0064	+0.0265
		6.252		N6···N9	0.0047	+0.0172
			**RCP**	(4 at.)	0.0064	+0.0273

[1] X: N,O or C
Y: N,O or C

Table S6. BSSE corrected interaction energies E_{int} and relative energies ΔE (kJ mol^{-1}) of the HNCO···N_2 complexes of the 2:1 stoichiometry calculated at MP2, B2PLYPD3 and B3LYPD3 levels with the aug-cc-pVTZ basis set.

Complex	MP2		B2PLYPD3		B3LYPD3	
	E_{int}	ΔE	E_{int}	ΔE	E_{int}	ΔE
2ON1	-26.02	0.00	-25.19	0.00	-25.15	0.00
2ON2	-25.27	0.88	-24.64	0.65	-25.10	0.18
2ON3	-24.35	1.82	-23.22	2.10	-23.77	1.51
2ON4	-23.60	2.43	-23.18	2.01	-24.31	0.88
2ON5	-21.88	4.44	-22.43	3.01	-22.93	2.48
2ON6	-21.34	4.62	-21.00	4.07	-21.92	3.15
2ON7	-20.59	5.36	-20.00	5.07	-20.96	4.11
2ON8	-20.04	5.88	-19.16	5.92	-20.88	4.21
2ON9	-19.04	6.93	-18.83	6.28	-19.71	5.35
2ON10	-17.74	8.17	-17.36	7.71	-18.70	6.39

Table S7. Theoretical infrared wavenumbers ($\bar{\upsilon}$, cm^{-1}), wavenumber shifts ($\Delta\bar{\upsilon}$, cm^{-1}) and intensities (I, km mol^{-1}) for monomers and 1:1 complexes using the MP2, B2PLYPD3 and B3LYPD3 methods with basis sets 6-311++G(3df,3pd) and aug-cc-pVTZ.

Mode	ON1			ON2			ON3			HNCO		N_2	
	$\bar{\upsilon}$	$\Delta\bar{\upsilon}$	I	$\bar{\upsilon}$	$\Delta\bar{\upsilon}$	I	$\bar{\upsilon}$	$\Delta\bar{\upsilon}$	I	$\bar{\upsilon}$	I	$\bar{\upsilon}$	I
MP2/6-311++G(3df,3pd)													
δNCO	592	21	76	568	-3	83	565	-6	97	571	94		
γNCO	642	6	0	636	0	1	636	0	1	636	1		
δHNC	789	4	239	795	10	211	794	9	233	785	215		
ν_sNCO	1308	2	1	1305	-1	0	1305	-1	0	1306	0		
νN≡N	2198	3	1	2193	-2	0	2194	-1	0			2195	0
ν_{as}NCO	2339	2	706	2335	-2	631	2335	-2	636	2337	675		
νNH	3704	-25	405	3723	-6	169	3720	-9	163	3729	175		
B2PLYPD3/6-311++G(3df,3pd)													
δNCO	596	22	63	572	-3	72	571	-4	81	575	79		
γNCO	640	6	1	635	0	2	635	0	2	635	2		
δHNC	809	6	231	812	9	209	816	13	233	803	213		
ν_sNCO	1317	2	2	1314	-1	0	1314	-1	0	1315	0		
ν_{as}NCO	2320	2	732	2318	-1	654	2317	-2	654	2319	696		
νN≡N	2348	6	0	2343	1	0	2344	2	0			2342	0
νNH	3677	-25	401	3697	-5	155	3694	-8	149	3702	159		
B3LYPD3/6-311++G(3df,3pd)													
δNCO	596	20	67	573	-3	75				576	83		
γNCO	643	4	3	639	0	3				639	3		
δHNC	802	4	217	806	8	201				798	205		
ν_sNCO	1338	2	2	1335	-1	0				1336	0		
ν_{as}NCO	2332	2	798	2330	0	712				2330	757		
νN≡N	2453	7	1	2447	1	0						2446	0
νNH	3658	-23	392	3677	-4	155				3681	159		

Mode	ON1			ON2			ON3			HNCO		N_2	
	$\bar{\upsilon}$	$\Delta\bar{\upsilon}$	I	$\bar{\upsilon}$	$\Delta\bar{\upsilon}$	I	$\bar{\upsilon}$	$\Delta\bar{\upsilon}$	I	$\bar{\upsilon}$	I	$\bar{\upsilon}$	I
MP2/aug-cc-pVTZ													
δNCO	591	21	68	567	-3	75	565	-5	88	570	85		
γNCO	636	7	0	630	1	0	630	1	0	629	0		
δHNC	799	4	244	805	10	215	804	9	239	795	220		
ν_sNCO	1300	1	1	1297	-2	0	1298	-1	0	1299	0		
νN≡N	2189	2	1	2184	-3	0	2185	-2	0			2187	0
ν_{as}NCO	2320	2	697	2317	-1	621	2317	-1	626	2318	666		
νNH	3674	-28	414	3695	-7	167	3693	-9	161	3702	172		
B2PLYPD3/aug-cc-pVTZ													
δNCO	595	21	59	571	-3	68	570	-4	77	574	75		
γNCO	638	6	1	632	0	1	632	0	2	632	2		
δHNC	813	6	234	816	9	211	819	12	236	807	216		
ν_sNCO	1313	2	2	1310	-1	0	1310	-1	0	1311	0		
ν_{as}NCO	2309	2	730	2306	-1	650	2306	-1	651	2307	693		
νN≡N	2347	6	0	2342	1	0	2343	2	0			2341	0
νNH	3664	-26	407	3686	-4	154	3683	-7	148	3690	159		
B3LYPD3/aug-cc-pVTZ													
δNCO	595	20	65	572	-3	72				575	80		
γNCO	642	5	2	638	1	3				637	3		
δHNC	804	5	219	808	9	203				799	206		
ν_sNCO	1336	2	2	1333	-1	0				1334	0		
ν_{as}NCO	2324	2	799	2322	0	712				2322	758		
νN≡N	2454	7	1	2449	2	0						2447	0
νNH	3651	-23	396	3670	-4	155				3674	159		

Table S8. Selected wavenumber shifts (cm^{-1}) calculated for the 1:1 complexes using the MP2, B2PLYPD3 and B3LYPD3 methods with the 6-311++G(3df,3pd) basis set. The shifts were calculated relative to the values obtained for the corresponding HNCO···Ar complexes. The calculated intensities (km mol^{-1}) of the bands are given in parentheses.

MP2			B2PLYPD3			B3LYPD3		Mode
ON1	ON2	ON3	ON1	ON2	ON3	ON1	ON2	
-19	-5	-6	-18	-4	-6	-17	-3	νNH
(405)	(169)	(163)	(401)	(155)	(149)	(392)	(155)	
+2	-1	-2	+1	0	-1	+2	0	ν_{as}NCO
(706)	(631)	(636)	(732)	(654)	(654)	(798)	(712)	
+19	-1	-4	+18	-1	-3	+16	0	δNCO
(76)	(83)	(97)	(63)	(72)	(81)	(67)	(75)	

Table S9. Theoretical infrared wavenumbers (ῡ, cm^{-1}), wavenumber shifts (Δῡ, cm^{-1}) and intensities (I, km mol^{-1}) for 1:2 complexes using the MP2, B2PLYPD3 and B3LYPD3 methods with basis sets 6-311++G(3df,3pd) and aug-cc- pVTZ.

Mode	O2N1			O2N2			O2N3			O2N4			O2N1			O2N2			O2N3			O2N4		
	ῡ	Δῡ	I	ῡ	Δῡ	I	ῡ	Δῡ	I	ῡ	Δῡ	I	ῡ	Δῡ	I	ῡ	Δῡ	I	ῡ	Δῡ	I	ῡ	Δῡ	I
	MP2/6-311++G(3df,3pd)												MP2/aug-cc-pVTZ											
δNCO	587	16	73	593	22	81	590	19	69	565	-6	96	586	16	64	592	22	73	588	18	61	564	-6	87
γNCO	642	6	0	643	7	0	642	6	0	635	-1	1	636	7	0	637	8	0	636	7	0	629	0	1
δHNC	800	15	241	793	8	268	799	14	232	795	10	235	810	15	245	803	8	276	808	13	235	805	10	241
ν_{s}NCO	1307	1	1	1307	1	1	1307	1	1	1305	-1	0	1300	1	1	1300	1	1	1299	0	1	1298	-1	0
νN≡N	2192	-3	0	2193	-2	0	2193	-2	0	2191	-4	0	2183	-4	0	2185	-2	0	2184	-3	0	2183	-4	0
νN≡N	2196	1	1	2197	2	1	2197	2	1	2192	-3	0	2188	1	1	2188	1	1	2189	2	1	2183	-4	0
ν_{as}NCO	2337	0	662	2337	0	740	2337	0	661	2336	-1	604	2319	1	652	2319	1	732	2319	1	651	2317	-1	592
νNH	3694	-35	380	3696	-33	398	3698	-31	397	3718	-11	159	3664	-38	388	3664	-38	409	3668	-34	407	3690	-12	157
	B2PLYPD3/6-311++G(3df,3pd)												B2PLYPD3/aug-cc-pVTZ											
δNCO	592	17	61	596	21	68	594	19	58				591	17	57	595	21	65	593	19	54			
γNCO	641	6	1	641	6	1	641	6	1				638	6	1	639	7	1	638	6	1			
δHNC	820	17	240	812	9	260	818	15	224				824	17	241	817	10	264	822	15	225			
ν_{s}NCO	1316	1	2	1317	2	2	1316	1	1				1312	1	2	1313	2	2	1312	1	2			
ν_{as}NCO	2319	0	687	2319	0	769	2319	0	688				2308	1	683	2308	1	767	2308	1	685			
νN≡N	2344	2	0	2344	2	0	2343	1	0				2343	2	0	2343	2	0	2342	1	0			
νN≡N	2347	5	0	2348	6	0	2348	6	0				2346	5	0	2347	6	0	2347	6	0			
νNH	3670	-32	376	3671	-31	391	3674	-28	394				3657	-33	383	3657	-33	400	3660	-30	401			
	B3LYPD3/6-311++G(3df,3pd)												B3LYPD3/aug-cc-pVTZ											
δNCO	592	16	66	592	16	78	593	17	62				592	17	63	594	19	72	593	18	59			
γNCO	644	5	2	644	5	3	644	5	2				642	5	2	643	6	2	642	5	2			
δHNC	814	16	229	805	7	248	810	12	211				815	16	230	807	8	249	812	13	213			
ν_{s}NCO	1337	1	2	1338	2	1	1337	1	2				1335	1	2	1336	2	2	1335	1	2			
ν_{as}NCO	2330	0	748	2330	0	837	2331	1	751				2323	1	747	2323	1	843	2323	1	751			
νN≡N	2449	3	0	2449	3	0	2447	1	0				2450	3	0	2450	3	0	2449	2	0			
νN≡N	2453	7	0	2452	6	0	2453	7	1				2454	7	1	2454	7	0	2454	7	1			
νNH	3652	-29	364	3658	-23	345	3655	-26	386				3645	-29	369	3647	-27	376	3648	-26	390			

Table S10. Theoretical infrared wavenumbers (ῡ, cm^{-1}), wavenumber shifts (Δῡ, cm^{-1}) and intensities (I, km mol^{-1}) for 2:1 complexes using the MP2, B2PLYPD3 and B3LYPD3 methods with basis sets 6-311++G(3df,3pd) and aug-cc-pVTZ.

Mode	2ON1			2ON2			2ON3			2ON4			2ON5			2ON6			2ON7			2ON8			2ON9			2ON10		
	ῡ	Δῡ	I	ῡ	Δῡ	I	ῡ	Δῡ	I	ῡ	Δῡ	I	ῡ	Δῡ	I	ῡ	Δῡ	I	ῡ	Δῡ	I	ῡ	Δῡ	I	ῡ	Δῡ	I	ῡ	Δῡ	I
	MP2/6-311++G(3df,3pd)																													
δNCO	575	4	187	582	11	26	566	-5	72	564	-7	136	596	25	100	563	-8	144	569	-2	105	561	-10	217	569	-2	136	559	-12	200
δNCO	637	66	0	634	63	37	632	61	22	590	19	46	609	38	70	608	37	92	601	30	48	568	-3	0	600	29	76	567	-4	7
γNCO	640	4	12	637	1	1	633	-3	1	635	-1	0	641	5	0	633	-3	1	634	-2	2	634	-2	0	635	-1	1	634	-2	0
γNCO	652	16	0	650	14	0	651	15	0	641	5	0	647	11	0	646	10	0	646	10	0	635	-1	2	646	10	0	636	0	1
δHNC	799	14	462	819	34	106	817	32	252	775	-10	538	748	-37	345	754	-31	378	752	-33	184	767	-18	517	746	-39	277	774	-11	518
δHNC	882	97	213	873	88	498	880	95	254	801	16	1	800	15	242	806	21	229	805	20	261	793	8	0	814	29	261	802	17	12
ν_sNCO	1305	-1	0	1304	-2	1	1301	-5	2	1307	1	2	1309	3	4	1308	2	3	1308	2	2	1308	2	3	1307	1	2	1306	0	1
ν_sNCO	1307	1	6	1307	1	6	1307	1	5	1309	3	3	1317	11	11	1314	8	5	1313	7	3	1308	2	0	1314	8	4	1308	2	1
νN≡N	2197	2	1	2195	0	1	2191	-4	0	2197	2	1	2198	3	1	2193	-2	0	2191	-4	0	2187	-8	0	2192	-3	0	2193	-2	0
ν_{as}NCO	2331	-6	1090	2330	-7	1553	2328	-9	650	2324	-13	9	2337	0	1329	2336	-1	1401	2332	-5	715	2327	-10	1	2334	-3	1298	2324	-13	0
ν_{as}NCO	2342	5	486	2342	5	21	2342	5	697	2348	11	1239	2350	13	321	2346	9	169	2349	12	659	2347	10	1109	2346	9	292	2346	9	1155
νNH	3584	-145	769	3579	-150	770	3576	-153	784	3681	-48	385	3658	-71	827	3656	-73	618	3658	-71	571	3712	-17	0	3660	-69	618	3707	-22	136
νNH	3665	-64	361	3668	-61	340	3690	-39	177	3709	-20	201	3705	-24	422	3732	3	186	3735	6	194	3713	-16	346	3739	10	209	3713	-16	212
	B2PLYPD3/6-311++G(3df,3pd)																													
δNCO	584	9	145	588	13	20	572	-3	60	569	-6	108	598	23	72	568	-7	107	571	-4	90	566	-9	171	571	-4	108	564	-11	160
δNCO	636	61	1	637	62	2	632	57	2	594	19	33	614	39	62	613	38	78	607	32	37	574	-1	0	606	31	65	573	-2	5
γNCO	644	9	11	638	3	32	635	0	19	634	-1	1	640	5	1	632	-3	3	634	-1	4	632	-3	0	634	-1	2	633	-2	0
γNCO	651	16	0	648	13	0	650	15	0	639	4	1	646	11	0	645	10	0	645	10	0	635	0	5	645	10	0	635	0	3
δHNC	821	18	442	842	39	112	837	34	249	799	-4	541	768	-35	329	775	-28	373	769	-34	186	790	-13	528	764	-39	273	796	-7	534
δHNC	905	102	215	897	94	470	901	98	244	826	23	3	824	21	247	829	26	235	828	25	254	816	13	0	836	33	266	824	21	8
ν_sNCO	1315	0	0	1314	-1	0	1311	-4	1	1316	1	2	1318	3	5	1317	2	3	1317	2	3	1316	1	4	1316	1	4	1315	0	2
ν_sNCO	1317	2	7	1317	2	8	1317	2	6	1318	3	3	1325	10	14	1322	7	7	1322	7	5	1317	2	0	1323	8	6	1316	1	1
ν_{as}NCO	2312	-7	1103	2311	-8	1599	2309	-10	679	2306	-13	7	2318	-1	1455	2317	-2	1510	2313	-6	772	2308	-11	1	2316	-3	1420	2305	-14	0
ν_{as}NCO	2326	7	532	2325	6	32	2326	7	724	2330	11	1303	2332	13	242	2328	9	99	2331	12	653	2329	10	1154	2328	9	208	2328	9	1209
νN≡N	2349	7	0	2347	5	0	2344	2	0	2349	7	1	2349	7	0	2345	3	1	2344	2	2	2340	-2	0	2344	2	0	2343	1	1
νNH	3548	-154	780	3545	-157	762	3544	-158	780	3656	-46	383	3622	-80	818	3622	-80	624	3626	-76	579	3687	-15	0	3628	-74	619	3683	-19	121
νNH	3638	-64	367	3642	-60	339	3666	-36	163	3684	-18	181	3677	-25	434	3705	3	172	3709	7	180	3688	-14	318	3712	10	192	3687	-15	200

	B3LYPD3/6-311++G(3df,3pd)																													
δNCO	585	10	137	589	14	22	575	-1	60	568	-8	112	597	22	80	566	-10	108	570	-6	96	567	-9	178	570	-6	115	565	-11	167
δNCO	639	64	2	640	65	3	636	61	4	594	19	35	618	43	68	617	42	84	609	34	39	574	-2	0	609	34	70	573	-3	5
γNCO	650	11	11	645	6	28	642	3	19	637	-2	2	644	5	3	638	-1	5	638	-1	7	636	-3	0	639	0	4	636	-3	0
γNCO	654	15	0	652	13	0	653	14	0	643	4	4	650	11	1	648	9	1	649	10	0	639	0	8	648	9	1	640	1	6
δHNC	818	20	394	837	39	113	834	36	238	796	-2	525	761	-37	305	773	-25	365	764	-34	171	788	-10	513	758	-40	252	794	-4	522
δHNC	902	104	229	894	96	459	896	98	243	824	26	2	820	22	246	825	27	219	823	25	248	816	18	0	835	37	262	823	25	6
ν_{s}NCO	1336	0	0	1335	-1	0	1333	-3	1	1336	0	3	1339	3	6	1339	3	3	1338	2	3	1337	1	4	1337	1	4	1336	0	3
ν_{s}NCO	1339	3	9	1338	2	9	1338	2	7	1339	3	3	1345	9	14	1342	6	7	1343	7	5	1338	2	0	1343	7	6	1336	0	0
ν_{as}NCO	2323	-7	1145	2322	-8	1738	2321	-9	734	2316	-14	8	2328	-2	1654	2327	-3	1671	2323	-7	825	2318	-12	2	2326	-4	1567	2315	-15	0
ν_{as}NCO	2339	9	635	2338	8	43	2339	9	799	2342	12	1424	2342	12	181	2338	8	63	2343	13	717	2341	11	1253	2339	9	189	2341	11	1319
νN≡N	2454	8	1	2453	7	1	2450	4	0	2455	9	1	2454	8	1	2451	5	1	2450	4	0	2445	-1	0	2449	3	0	2448	2	0
νNH	3511	-170	830	3507	-174	810	3510	-171	808	3637	-44	369	3593	-88	804	3596	-85	625	3601	-80	571	3666	-15	0	3602	-79	606	3662	-19	112
νNH	3621	-60	344	3628	-53	315	3647	-34	163	3661	-20	181	3657	-24	446	3683	2	171	3689	8	182	3667	-14	316	3691	10	194	3665	-16	207

Mode	2ON1			2ON2			2ON3			2ON4			2ON5			2ON6			2ON7			2ON8			2ON9			2ON10		
	$\bar{\upsilon}$	$\Delta\bar{\upsilon}$	I	$\bar{\upsilon}$	$\Delta\bar{\upsilon}$	I	$\bar{\upsilon}$	$\Delta\bar{\upsilon}$	I	$\bar{\upsilon}$	$\Delta\bar{\upsilon}$	I	$\bar{\upsilon}$	$\Delta\bar{\upsilon}$	I	$\bar{\upsilon}$	$\Delta\bar{\upsilon}$	I	$\bar{\upsilon}$	$\Delta\bar{\upsilon}$	I	$\bar{\upsilon}$	$\Delta\bar{\upsilon}$	I	$\bar{\upsilon}$	$\Delta\bar{\upsilon}$	I	$\bar{\upsilon}$	$\Delta\bar{\upsilon}$	I
	MP2/aug-cc-pVTZ																													
δNCO	575	5	173	581	11	25	565	-5	64	563	-7	123	594	24	85	563	-7	127	567	-3	96	561	-9	194	568	-2	121	559	-11	178
δNCO	631	61	0	631	61	33	626	56	1	589	19	40	607	37	67	606	36	84	599	29	43	568	-2	0	597	27	69	567	-3	7
γNCO	637	8	10	632	3	0	629	0	19	629	0	0	636	7	0	627	-2	1	628	-1	1	628	-1	0	629	0	1	628	-1	0
γNCO	647	18	0	645	16	1	647	18	1	635	6	0	642	13	0	641	12	0	640	11	0	629	0	1	640	11	0	629	0	1
δHNC	808	13	481	829	34	110	827	32	253	784	-11	555	755	-40	361	762	-33	390	759	-36	190	776	-19	534	752	-43	287	783	-12	533
δHNC	894	99	212	886	91	495	893	98	254	810	15	0	809	14	246	815	20	238	815	20	260	803	8	0	824	29	266	812	17	14
ν_{s}NCO	1298	-1	0	1297	-2	1	1294	-5	1	1300	1	2	1301	2	4	1300	1	3	1300	1	2	1300	1	3	1300	1	3	1299	0	2
ν_{s}NCO	1300	1	6	1300	1	7	1299	0	5	1302	3	3	1309	10	12	1306	7	5	1306	7	3	1301	2	0	1307	8	5	1300	1	1
νN≡N	2188	1	1	2186	-1	1	2182	-5	0	2188	1	1	2190	3	1	2184	-3	0	2182	-5	0	2178	-9	0	2184	-3	0	2184	-3	0
ν_{as}NCO	2312	-6	1082	2312	-6	1538	2310	-8	638	2305	-13	10	2319	1	1318	2317	-1	1373	2314	-4	698	2308	-10	1	2316	-2	1298	2304	-14	0
ν_{as}NCO	2324	6	481	2324	6	23	2324	6	691	2330	12	1216	2332	14	315	2327	9	176	2330	12	656	2329	11	1080	2328	10	278	2329	11	1128
νNH	3546	-156	798	3541	-161	797	3536	-166	813	3649	-53	400	3623	-79	845	3621	-81	635	3624	-78	583	3683	-19	0	3627	-75	634	3678	-24	135
νNH	3635	-67	365	3638	-64	348	3663	-39	176	3681	-21	198	3673	-29	438	3704	2	184	3708	6	193	3684	-18	340	3712	10	208	3684	-18	208

B2PLYPD3/aug-cc-pVTZ																														
δNCO	583	9	140	588	14	19	571	-3	57	568	-6	103	597	23	66	567	-7	101	571	-3	86	565	-9	162	571	-3	102	564	-10	151
δNCO	634	60	1	634	60	1	629	55	2	593	19	30	613	39	60	612	38	74	605	31	35	573	-1	0	604	30	61	572	-2	5
γNCO	642	10	10	636	4	29	634	2	17	631	-1	1	638	6	1	630	-2	3	631	-1	4	629	-3	0	631	-1	2	630	-2	0
γNCO	648	16	0	646	14	0	648	16	0	637	5	1	644	12	0	642	10	0	643	11	0	632	0	4	642	10	0	632	0	3
δHNC	825	18	453	847	40	114	842	35	251	802	-5	545	771	-36	335	778	-29	377	772	-35	189	793	-14	533	766	-41	279	800	-7	537
δHNC	911	104	214	903	96	468	907	100	244	829	22	3	828	21	248	833	26	239	832	25	254	820	13	0	840	33	268	828	21	9
ν_sNCO	1311	0	0	1310	-1	0	1308	-3	1	1312	1	3	1314	3	5	1314	3	3	1313	2	3	1312	1	4	1313	2	4	1311	0	3
ν_sNCO	1313	2	8	1313	2	8	1313	2	7	1314	3	4	1321	10	15	1318	7	8	1318	7	5	1313	2	0	1318	7	7	1312	1	1
ν_{as}NCO	2301	-6	1107	2300	-7	1599	2298	-9	677	2295	-12	7	2307	0	1450	2306	-1	1498	2302	-5	766	2296	-11	1	2305	-2	1425	2294	-13	0
ν_{as}NCO	2315	8	527	2314	7	29	2315	8	720	2319	12	1295	2321	14	243	2317	10	105	2321	14	654	2318	11	1142	2317	10	201	2318	11	1199
νN≡N	2348	7	1	2346	5	0	2343	2	0	2348	7	1	2348	7	1	2344	3	1	2343	2	0	2339	-2	0	2343	2	0	2342	1	0
νNH	3530	-160	794	3527	-163	777	3526	-164	797	3642	-48	392	3606	-84	835	3606	-84	637	3610	-80	589	3676	-14	0	3613	-77	631	3672	-18	122
νNH	3625	-65	370	3629	-61	345	3654	-36	163	3673	-17	180	3663	-27	442	3694	4	172	3698	8	180	3676	-14	317	3700	10	192	3676	-14	197
B3LYPD3/aug-cc-pVTZ																														
δNCO	585	10	136	589	14	22	574	-1	58	567	-8	110	596	21	76	565	-10	105	570	-5	94	566	-9	173	570	-5	112	564	-11	161
δNCO	638	63	2	638	63	3	634	59	3	593	18	34	617	42	66	616	41	81	608	33	37	574	-1	0	608	33	68	572	-3	5
γNCO	648	11	10	644	7	26	640	3	17	636	-1	1	643	6	3	636	-1	4	637	0	6	634	-3	0	637	0	3	635	-2	0
γNCO	652	15	0	651	14	0	652	15	0	641	4	3	648	11	0	647	10	1	647	10	0	638	1	7	647	10	1	638	1	5
δHNC	819	20	403	840	41	116	835	36	240	797	-2	527	762	-37	309	774	-25	366	765	-34	174	789	-10	515	759	-40	258	795	-4	523
δHNC	905	106	227	897	98	458	900	101	243	825	26	2	822	23	247	827	28	223	824	25	248	817	18	0	836	37	263	824	25	8
ν_sNCO	1334	0	0	1333	-1	0	1331	-3	1	1334	0	3	1337	3	6	1336	2	3	1336	2	4	1335	1	4	1335	1	4	1333	-1	3
ν_sNCO	1336	2	9	1336	2	10	1336	2	8	1336	2	4	1342	8	15	1339	5	8	1340	6	5	1335	1	0	1340	6	7	1334	0	0
ν_{as}NCO	2315	-7	1155	2314	-8	1748	2312	-10	736	2308	-14	9	2320	-2	1652	2319	-3	1661	2315	-7	825	2310	-12	2	2318	-4	1581	2307	-15	0
ν_{as}NCO	2331	9	630	2330	8	36	2331	9	797	2334	12	1423	2334	12	186	2330	8	73	2335	13	718	2333	11	1248	2331	9	181	2333	11	1317
νN≡N	2456	9	1	2454	7	1	2452	5	0	2456	9	1	2455	8	1	2453	6	1	2451	4	0	2446	-1	0	2451	4	0	2449	2	0
νNH	3500	-174	838	3496	-178	819	3499	-175	820	3630	-44	376	3583	-91	819	3586	-88	634	3592	-82	582	3660	-14	1	3594	-80	617	3656	-18	116
νNH	3614	-60	344	3620	-54	320	3641	-33	163	3655	-19	181	3649	-25	452	3677	3	172	3683	9	182	3661	-13	316	3685	11	195	3659	-15	204
